# Postoperative structured nursing plan for patients with cervical spine fracture and spinal cord injury: a retrospective study

**DOI:** 10.3389/fsurg.2026.1928795

**Published:** 2026-09-01

**Authors:** Ni Luo, Aijun Xu, Shanshan Xu, Bo Chen, Yunlin Chen, Xudong Hu, Jing Li

**Affiliations:** 1 Department of Operating Room Nursing, Ningbo No.6 Hospital, Ningbo, Zhejiang, China; 2 Ningbo Clinical Research Center for Orthopedics, Sports Medicine & Rehabilitation, Ningbo, Zhejiang, China

**Keywords:** cervical spine fracture, perioperative period, psychological state, spinal cord injury, structured nursing

## Abstract

**Objective:**

This study aimed to examine the efficacy of structured nursing in promoting postoperative recovery among patients with cervical spine fracture and spinal cord injury.

**Methods:**

This study is a retrospective cohort analysis. A total of 100 patients with cervical spine fracture and spinal cord injury who underwent anterior cervical discectomy and fusion (ACDF) at our Hospital from December 2023 to December 2025 were included as the research subjects. They were divided into control group (Routine nursing, *n* = 55) and observation group (Structured nursing, *n* = 45) according to actual nursing measures. Patients in the control group received routine care, while those in the observation group received structured nursing. Patients in both groups received nursing intervention until discharge. A comprehensive comparison was conducted between the two groups, covering the following aspects: psychological state, living function, motor function, cervical spine function, pain degree, quality of life, the incidence of complications during hospitalization, clinical efficacy, and nursing satisfaction.

**Results:**

After patient care to discharge from hospital, the Self-Rating Depression Scale (SDS) and Self-Rating Anxiety Scale (SAS), Activities of Daily Living (ADL), and VAS scores in the structured nursing group were significantly lower than those in the normal group; while the FMA, JOA scores were significantly higher than those in the normal group. After nursing, the psychological, social, physical and material function scores of patients in both groups were higher than those before nursing, and the structured nursing group was higher than those in the normal group. The overall incidence of complications during hospitalization in the structured nursing group was 13.33%, which was significantly lower than that in the Routine nursing group (38.18%). Patients receiving structured nursing had better clinical outcomes and higher nursing satisfaction relative to the normal group.

**Conclusion:**

Structured nursing can enhance the treatment outcome for patients with cervical spine fracture and spinal cord injury, alleviate pain and improve their negative emotions, and can also reduce the incidence of complications.

## 1 Introduction

Patients with cervical spine fracture have severe injuries, most of whom are accompanied by spinal cord injury. The main clinical symptoms of patients include neck pain, limited mobility, neck muscle spasm and widespread neck tenderness (1). Currently, surgical treatment is mainly used for the clinical treatment of this disease. However, such patients have a long postoperative recovery period and have limb motor dysfunction after surgery, which has a significant impact on the patient's normal activities and easily causes serious negative emotions, which may impede the patient's recovery process (2). Research indicates that postoperative care can effectively alleviate the patient's pain, improve the stability of the cervical spine, and reduce postoperative complications (3). Currently, clinical practice mostly adopts routine care, which promotes physical recovery by monitoring patients’ physiological indicators, guiding patients to take medication, and exercising (4). Although conventional nursing methods have a certain effect, they lack targetedness in implementation and insufficient attention to the patients’ psychological state, ignoring the psychological needs of the patients during treatment, resulting in poor patient compliance and thus affecting the long-term therapeutic effect. Therefore, adopting scientific and reasonable nursing intervention measures and combining nursing with humanistic care is of great significance for the functional recovery of the body and the improvement of postoperative quality of life of patients with cervical spine fracture and spinal cord injury (5, 6).

Compared with routine care, structured nursing, as a new type of nursing approach gradually promoted in clinical practice, is characterized by integrating multiple nursing methods into every aspect of the work. It ensures that each aspect can be dataized and refined, and avoiding any loopholes in the process of nursing intervention and dynamic monitoring of the patient's condition. This nursing model helps nursing staff implement comprehensive, scientific, and standardized nursing intervention, achieving more ideal nursing outcomes (7, 8). In order to evaluate the efficacy of structured nursing in postoperative rehabilitation for patients with cervical spine fracture and spinal cord injury, this study retrospectively analyzed the clinical data of patients with cervical spine fracture and spinal cord injury who underwent anterior cervical discectomy and fusion (ACDF) at our Hospital from December 2023 to December 2025. The analysis results demonstrated the significant advantages of structured nursing over routine care. The specific results are now reported as follows.

## 2 Materials and methods

### 2.1 Clinical data

This study included a total of 100 patients with cervical spine fracture and spinal cord injury who underwent ACDF at our Hospital from December 2023 to December 2025 as the research subjects. Inclusion criteria: (1) diagnosis of cervical spine fracture with spinal cord injury confirmed by imaging examinations such as CT or MRI; (2) clear consciousness without cognitive impairment; (3) Frankel spinal cord injury classification grades A-D; (4) availability of complete clinical data. Exclusion criteria: (1) patients with a history of cervical spine fracture; (2) patients with multiple fractures; (3) patients with severe chronic diseases such as hypertension and diabetes; (4) patients with mental disorders or blood diseases. Patients were divided into control group (Normal, *n* = 55) and observation group (Structured nursing, *n* = 45) according to actual nursing measures. The baseline clinical data showed no statistically significant differences between the two groups (*P* > 0.05), indicating comparability (Table 1).

**Table 1:** Comparison of general information of patients between the two groups.

Item	Routine nursing (*n* = 55)	Structured nursing (*n* = 45)	*t*/χ^2^	*P* value
Age (years)	50.02 ± 7.34	50.62 ± 6.60	0.428	0.670
Gender			0.000	0.984
Male	28	23		
Female	27	22		
BMI (kg/m^2^)	25.12 ± 2.69	25.02 ± 3.01	0.171	0.865
Cause of injury			0.160	0.984
Heavy object injury	16 (29.09)	13 (28.89)		
Traffic accident	22 (40.00)	18 (40.00)		
Fall from height	11 (20.00)	10 (22.22)		
Other	6 (10.91)	4 (8.89)		
Frankel classification			0.060	0.996
A	20 (36.36)	17 (37.78)		
B	16 (29.09)	13 (28.89)		
C	12 (21.82)	9 (20.00)		
D	7 (12.73)	6 (13.33)		

### 2.2 Nursing methods

Routine care was administered to the patients in the control group. The nursing staff monitored the changes in vital signs during the recovery period, guided patients to follow doctor's instructions for medication, assisted them in completing postoperative rehabilitation training after their conditions stabilized, and explained daily life precautions to the patients before their discharge. Structured nursing was applied to the patients in the observation group. The specific nursing measures are as follows: (1) A structured rehabilitation nursing team was established: its main members including quality control officers, primary nurses, ward head nurses, and department head nurses. All participating members were required to have rich theoretical knowledge in nursing and solid clinical nursing skills, and be familiar with the practical methods of cervical spine fracture with spinal cord injury rehabilitation nursing, as well as information retrieval methods. (2) A structured nursing plan was developed: the overall plan for the patient's structured nursing was determined, with the basic nursing model being constituted by the inclusion of medication guidance, dietary guidance, pain care, safety care, psychological care, cognitive intervention, rehabilitation training, and complication prevention. (3) Training on the structured nursing plan: the structured nursing plan was compiled into a presentation to train nursing staff, enabling them to master the details and basis of the nursing plan. All members were assessed to ensure that every nurse grasps the essentials of structured nursing in a systematic, comprehensive and holistic manner. (4) During the operation, we assigned a dedicated neurophysiological monitoring nurse to monitor the patient's neurological functions while they were under anesthesia. (5) Implementation of the structured nursing plan: 1. Medication guidance. The importance of medication adherence was emphasized to the patient, as well as the use of various drugs and adverse drug reactions. The patient's medication intake was closely monitored for any instances of irregular dosing, overuse, underuse, or non-adherence. 2. Dietary guidance. The patient's previous dietary habits were reviewed, and the impact of unhealthy eating on the disease was explained. Based on the patient's condition, a diet high in protein, vitamins, calories, and calcium, along with easily digestible semi-liquid foods, was gradually introduced to enhance nutritional intake. 3. Pain care. The patient's pain status was regularly assessed, and targeted pain control measures were adopt based on the patient's pain. If the pain was severe, the doctor was promptly notified for the administration of analgesic medications. 4. Safety care. The severity of the patient's condition and risk of falls were assessed. The potential risk of falls in both outdoor and hospital environments was clearly clarified to the patient and their family, and targeted, individualized fall prevention measures were developed. 5. Psychological care. Some patients have serious negative emotions, even resistance to rehabilitation treatment. The primary nurses should cooperate with psychologists to conduct targeted communication with patients, actively understand the patient's inner world, listen to their true demands, encourage them to vent their negative emotions, and adopt scientific and systematic psychological nursing methods to regulate their emotions and convey expectations for rehabilitation. 6. Cognitive intervention. The patients were educated about the postoperative recovery process for cervical spine fracture with spinal cord injury. Successful surgical treatment cases were shared to enhance patients’ disease comprehension, boost their confidence in overcoming it, and improve their self-care abilities. 7. Rehabilitation training. Phased rehabilitation goals were formulated: during the bedridden period, focus on preventing muscle atrophy and joint deformities by instructing the patient to wear orthopedic shoes; in the early stages of rehabilitation, assist the patient with passive exercises; once basic limb functions are restored, help the patient overcome psychological barriers and encourage them to actively engage in upper and lower limb exercises, lower back muscle exercises, sitting-up exercises, wheelchair transfer exercises, and walking exercises. 8. Prevention of complications. Closely monitor the patient's vital signs after surgery to ensure that the patient's respiratory tract is unobstructed. Instruct patients to effectively expectorate and breathe deeply, and if necessary, perform aerosol inhalation to facilitate expectoration and promote lung function recovery, preventing pulmonary infections; ensure that the negative pressure drainage tube is unobstructed and airtight, and keep the local dressing dry; regularly change the bed sheets, assist the patient in performing axial turning every two hours to maintain skin dryness, and massage the pressed areas of the bony processes, and use soft cushions to protect if necessary; instruct the patient to drink more water and keep the perineum and urethral orifice clean. For patients with indwelling urinary catheters, regularly replace the catheter and urine bags to prevent urinary tract infections; encourage and assist the patient to perform early lower limb activity exercises, provide passive movement and massage, to prevent deep vein thrombosis of the lower extremities. Patients in both groups were given nursing intervention until discharge.

Both group’s nursing care are neutrally, and each patient received care according to existing hospital protocols and that the structured nursing represents an enhanced, supplementary approach rather than a criticism of routine practice.

### 2.3 Observation indicators and evaluation criteria

1. Psychological state: Before and after intervention, the Self-Rating Depression Scale (SDS) and Self-Rating Anxiety Scale (SAS) were employed to assess patients’ psychological state. The standard cut-off values of SDS and SAS were 53 points and 50 points respectively. A lower score indicates a better psychological state.
2. Living function and motor function: Before and after nursing, the Activities of Daily Living (ADL) scale was used for the assessment of living function. The scale included physical self-care and instrumental daily living activities. There were 14 scoring items in total, with scores of 0–56 points. A higher score indicates poorer daily living ability; the Fugl-Meyer Assessment (FMA) scale was administered to evaluate motor function, which included 33 scoring items for the upper limbs and 17 scoring items for the lower limbs, with scores of 0–100. The higher the score, the better the motor function of the limbs.
3. Cervical spine function and pain severity: Before and after nursing, the patient's cervical spine function was assessed, specifically with the Japanese Orthopaedic Association (JOA) Scoring System for Cervical Myelopathy. The total score was 17 points. The higher the score, the better the patient's cervical spine function; the patient's pain severity was measured using the Visual Analogue Scale (VAS), with the score range of 0–10 points. A lower score indicates milder pain levels in patients.
4. Quality of life: Before and after intervention, quality of life was measured with the Generic Quality of Life Inventory-74 (GQOL-74). The content of this scale includes 4 dimensions: psychological, social, physical and material functions. Scores for each dimension range from 0 to 100, and higher scores represent a superior quality of life.
5. Incidence of complications: Various complications occurring during the intervention period were recorded for comparison between the two groups, mainly including incision infection, subcutaneous hematoma, pulmonary infection, deep vein thrombosis of the lower extremities, urinary tract infection and sacrococcygeal pressure sores.
6. Clinical efficacy and nursing satisfaction: According to the relevant literature (9), the therapeutic effect was classified into three grades: marked effect, effective, and ineffective. The total effective rate = (marked effect + effective) number of cases/total number of cases × 100%. A self-made questionnaire was used to survey patients’ satisfaction with the nursing service, which was divided into three grades: very satisfied, satisfied and dissatisfied. Nursing satisfaction = (very satisfied + satisfied) number of cases/total cases × 100%.

### 2.4 Statistical analysis

The data of this study were analyzed and processed using SPSS 24.0 statistical software. The measurement data were expressed as ($\bar{x}$± *s*) and compared using the *t*-test. The counting data were expressed as rate (%) and analyzed with the chi-square (*χ*^2^) test. A *P*-value of less than 0.05 was considered statistically significant.

## 3 Results

### 3.1 Comparison of psychological state of the two patient groups before and after nursing

**Figure 1:**
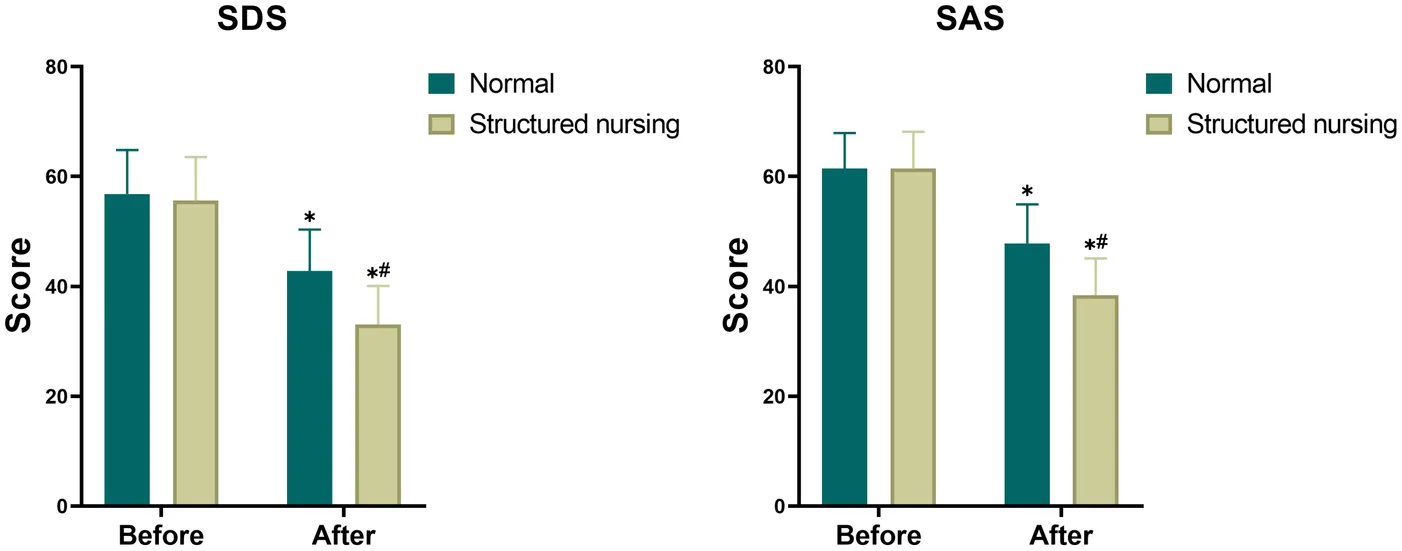
Comparison of SDS and SAS scores representing the psychological states (depression, anxiety) before and after nursing between the two groups of patients. *P < 0.05, compared with the same group before nursing; #*P* < 0.05, compared with the Routine nursing after nursing.

Prior to nursing intervention, SDS and SAS scores showed no significant baseline difference between the groups (*P* > 0.05). After nursing, the SDS and SAS scores of patients in both groups were significantly lower than those before nursing, and both scores in the structured nursing group were significantly lower than those in the normal group, with statistical significance (*P* < 0.05, Figure 1).

### 3.2 Comparison of living function and motor function of the two patient groups before and after nursing

**Figure 2:**
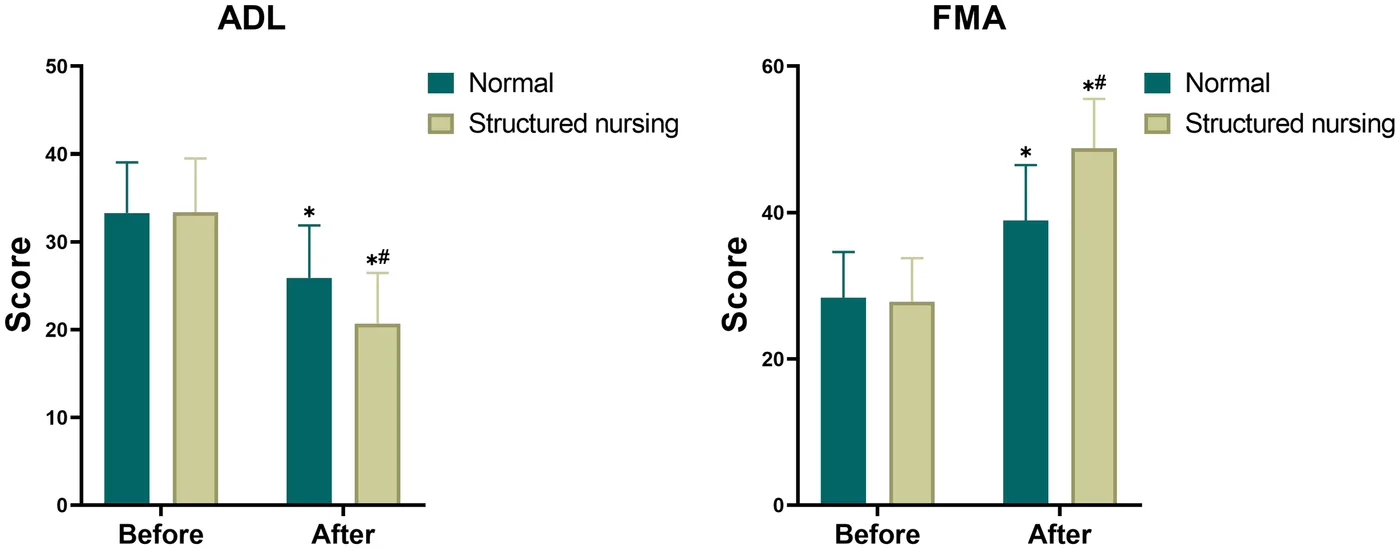
Comparison of ADL and FMA scores representing self-care ability and limb motor function between the two groups of patients before and after nursing. *P < 0.05, compared with the same group before nursing; #*P* < 0.05, compared with the Routine nursing after nursing.

Prior to nursing intervention, ADL and FMA scores showed no significant baseline difference between the groups (*P* > 0.05). After nursing, the ADL scores in both groups were significantly lower than before nursing, indicating that the patient's self-care ability was significantly improved after nursing; both groups demonstrated significant improvement in FMA scores following the nursing intervention, indicating that the patient's limb motor function and coordination were significantly improved after nursing. Among them, the improvement of self-care ability and limb motor function in patients in the structured nursing group was better than that in the Routine nursing group (*P* < 0.05, Figure 2).

### 3.3 Comparison of cervical spine function and pain severity of the two patient groups before and after nursing

**Figure 3:**
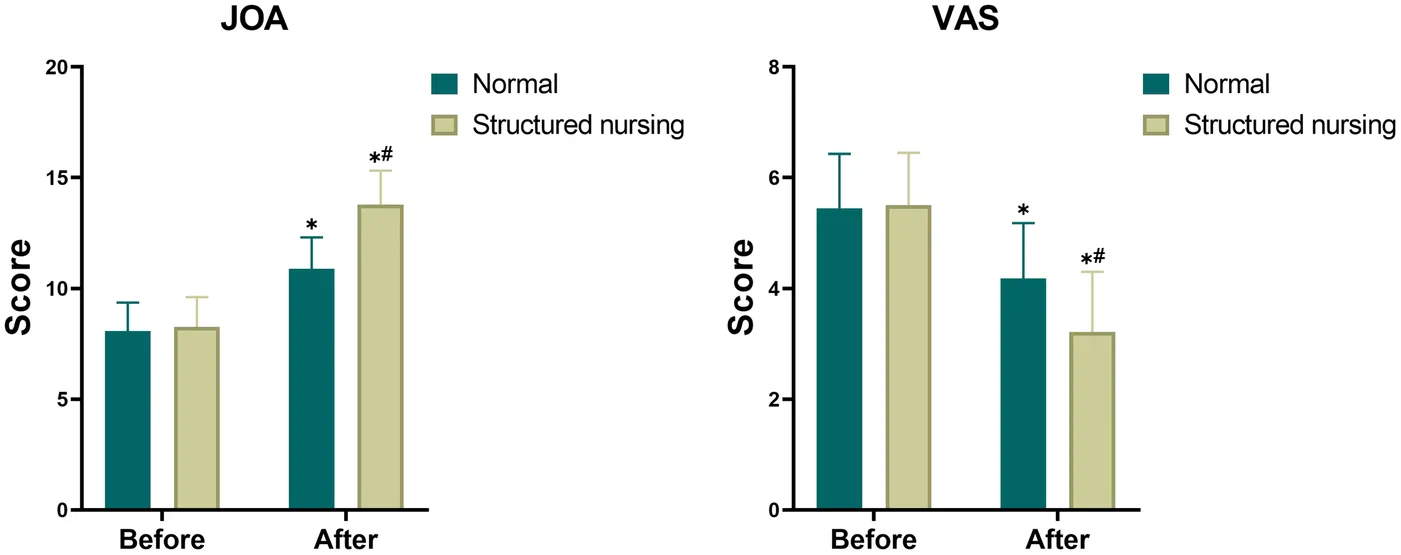
Comparison of JOA and VAS scores representing cervical spine function and pain degree before and after nursing between the two groups of patients. *P < 0.05, compared with the same group before nursing; #*P* < 0.05, compared with the Routine nursing after nursing.

Prior to nursing intervention, JOA and VAS scores showed no significant baseline difference between the groups (*P* > 0.05). After nursing, the JOA scores in both groups were significantly higher than before nursing, indicating that the patient's cervical spine function was effectively improved after receiving nursing intervention; the VAS scores in both groups were significantly lower than before nursing, indicating that the patient's pain level was effectively relieved after receiving nursing intervention. Among them, the improvement in cervical spine function and pain in patients in the structured nursing group was better than that in the Routine nursing group (*P* < 0.05, Figure 3).

### 3.4 Comparison of quality of life of the two patient groups before and after nursing

**Figure 4:**
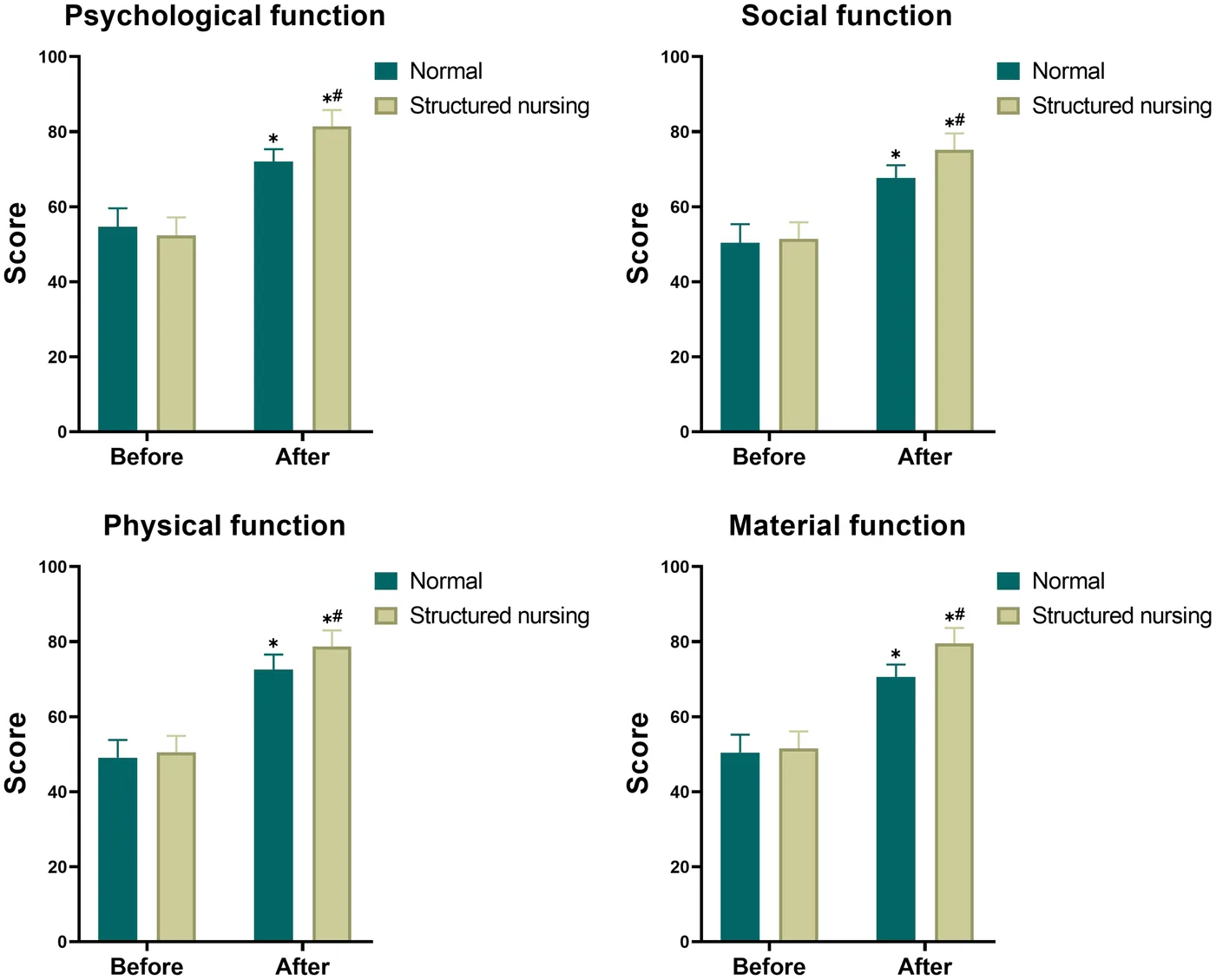
Comparison of the scores of the four dimensions of GQOL-74 between the two groups of patients before and after nursing. *P < 0.05, compared with the same group before nursing; #*P* < 0.05, compared with the Routine nursing after nursing.

Prior to nursing intervention, psychological, social, physical and material function scores showed no significant baseline difference between the groups (*P* > 0.05). After nursing, the psychological, social, physical and material function scores of patients in both groups were higher than those before nursing, and the structured nursing group was higher than that of the normal group, with statistical significance (*P* < 0.05, Figure 4).

### 3.5 Comparison of the incidence of complications between the two patient groups

The structured nursing group exhibited a significantly lower in the rate of postoperative complications (13.33%) than the Routine nursing group (38.18%) (*P* < 0.05, Table 2).

**Table 2:** Comparison of the incidence of complications between the two groups of patients [*n* (%)].

Group	Incision infection	Subcutaneous hematoma	Pulmonary infection	Deep vein thrombosis of the lower extremities	Urinary tract infection	Sacrococcygeal pressure sore	Total
Routine nursing (*n* = 55)	5 (9.09)	1 (1.82)	3 (5.45)	3 (5.45)	3 (5.45)	6 (10.91)	21 (38.18)
Structured nursing (*n* = 45)	1 (2.22)	0 (0.00)	1 (2.22)	1 (2.22)	1 (2.22)	2 (4.44)	6 (13.33)
*χ* ^2^	2.070	0.826	0.673	0.673	0.673	1.405	7.753
*P* value	0.150	0.363	0.412	0.412	0.412	0.236	0.005

### 3.6 Comparison of clinical efficacy and nursing satisfaction between the two patient groups

After intervention, in the normal group, 25 cases showed marked effect, 20 cases were effective, and 10 cases were ineffective, with a total treatment effective rate of 81.81%; in the structured nursing group, 28 cases showed marked effect, 15 cases were effective, and 2 cases were ineffective, with a total treatment effective rate of 95.55%. The total effective rate of the structured nursing group was higher than that of the normal group, with statistical significance (*P* < 0.05, Table 3). After intervention, the Routine nursing group reported an overall satisfaction rate of 78.18% (21 very satisfied, 22 satisfied, 12 dissatisfied), whereas the structured nursing group achieved a significantly higher rate of 95.55% (24 very satisfied, 19 satisfied, 2 dissatisfied); those difference was statistically significant (*P* < 0.05, Table 4).

**Table 3:** Comparison of clinical efficacy between the two groups [*n* (%)].

Group	Marked effect	Effective	Ineffective	Total effective
Routine nursing (*n* = 55)	25 (45.45)	20 (36.36)	10 (18.18)	45 (81.81)
Structured nursing (*n* = 45)	28 (62.22)	15 (33.33)	2 (4.44)	43 (95.55)
*χ* ^2^				4.423
*P* value				0.036

**Table 4:** Comparison of nursing satisfaction between the two groups [*n* (%)].

Group	Very satisfied	Satisfied	Dissatisfied	Nursing satisfaction
Normal (*n* = 55)	21 (38.18)	22 (40.00)	12 (21.82)	43 (78.18)
Structured nursing (*n* = 45)	24 (53.33)	19 (42.22)	2 (4.44)	43 (95.55)
*χ* ^2^				6.205
*P* value				0.013

## 4 Discussion

Surgery is the best solution for clinical treatment of cervical spine fracture with spinal cord injury. However, patients have a long postoperative recovery period and are prone to complications such as incision infection and deep vein thrombosis of the lower extremities. In addition, they are often accompanied by motor dysfunction and a decline in daily living abilities, causing the patient to have serious negative emotions (10). In order to improve the postoperative rehabilitation effect of patients with cervical spine fracture and spinal cord injury, effective nursing intervention is needed (11). Structured nursing is a comprehensive, systematic and scientific nursing model. Guided by the concept of holistic nursing, structured nursing is carried out through systematic, standardized and procedural forms. In recent years, structured rehabilitation nursing has had remarkable effects in postoperative recovery in orthopedic surgery. This nursing model can effectively eliminate the limitations of conventional nursing, such as the lack of targeting and standardization (12–14). This study compared the efficacy of routine care and structured nursing in patients with cervical spine fracture and spinal cord injury undergoing ACDF, and the results highlighted the superiority of structured nursing.

Due to cervical spine fracture accompanied by spinal cord injury, irreversible damage was caused to some neurological functions, resulting in the sudden loss of the patient's ability to live independently, causing the patient's negative emotions such as anxiety and depression. The patient's treatment compliance decreased, and even refusal of treatment occurred from time to time (10). In this study, the structured nursing group showed a greater reduction in SDS and SAS scores than the normal group. This indicates that under the intervention of structured nursing, the nursing staff fully recognized the relationship between emotions and rehabilitation, and deeply understood the patient's psychological problems, providing them with continuous positive encouragement, which could significantly improve the patient's emotional state and improve nursing compliance (15). To analyze the reasons, for patients with cervical spine fracture and spinal cord injury, the sudden and unpredictable occurrence of cervical fracture, post-fracture pain, loss of function, the uncertainty of the treatment process and prognosis, as well as economic pressure the main stressors that cause negative emotions, and they are also unfavorable factors affecting the quality of medical care. Psychological nursing in structured nursing is a comprehensive psychological intervention method based on holistic medicine. Psychological support is always adhered to during the nursing process, and targeted psychological intervention is given by analyzing stressors that affect psychology, avoiding the universality and uniqueness of routine care (16). The structured nursing group in this study improved the overall nursing level of the nursing staff by forming a professional nursing team, comprehensively and systematically grasped the physical and mental stress characteristics of patients with cervical spine fracture and spinal cord injury and the measures and procedures of structured psychological nursing, providing professional personnel guarantee for the psychological intervention of patients; in addition, the structured nursing group also greatly improved the patient's systematic understanding of cervical spine fracture through cognitive intervention, reduced the uncertainty of the disease, enabled patients to maintain a relatively stable emotional state during the perioperative period, cooperate actively with the medical staff, which was conducive to postoperative rehabilitation, and further alleviated patients’ anxiety and depression emotions. Furthermore, the ADL score and FMA score of patients in the structured nursing group after nursing were better than those in the normal group, suggesting that structured nursing can improve patients’ living and motor functions. This suggests that structured nursing continuously instills rehabilitation nursing knowledge, enabling the internalization of health beliefs, allowing the patients to fully recognize the role of postoperative nursing and actively cooperate with nursing intervention, thereby achieving comprehensive improvement in various physiological functions (17). The reason for this is that after the implementation of structured nursing, the head nurse and senior nursing staff used evidence-based nursing methods to retrieve, evaluate and structurally handle the related nursing care for patients with cervical spine fracture and spinal cord injury. They helped the nursing staff understand the implementation basis and purpose of various nursing items through collective interpretation, supported the nursing staff to organize nursing strategies in a clear and correct manner in the form of nursing modules, guided the nursing staff to quickly determine the specific types of nursing behaviors through the nursing measure linkage method, enhanced the nursing staff's practical ability through self-study and service scripts. On the one hand, when facing patients with cervical spine fracture and spinal cord injury, nurses have the ability to provide scientific and appropriate basis explanations for the nursing plans, guide patients to improve their active compliance on the basis of understanding and recognition, thereby cooperating actively with the guidance of the nursing staff for rehabilitation training, achieving better recovery effects in terms of living function and motor function (18). During the evaluation of the patients’ cervical function and pain improvement effect, it was found that after nursing to discharge, the JOA score of the patients in the structured nursing group was significantly higher than that before nursing and significantly higher than that of the normal group, and the VAS score was significantly lower than that before nursing and significantly lower than that of the normal group. This indicates that structured nursing can effectively improve the cervical function and pain degree of patients with cervical vertebra fracture and spinal cord injury. The advantage of structured nursing lies in that the members of the structured nursing team responsible for this group of nursing work apply the rehabilitation concept and the core idea of precise and demand-based nursing to key preoperative and postoperative nursing links of patients based on individual health needs. This helps enhance the patients’ and their families’ awareness of the benefits of functional exercise, stimulate their rehabilitation potential and enthusiasm, achieve the goals of precise education, precise nursing, and precise rehabilitation, and achieve effective recovery of cervical spine function; cooperating with safety care during the rehabilitation process can effectively avoid secondary trauma caused by improper protection; at the same time, implementing pain care can help patients reduce pain and relieve postoperative physical and mental stress reactions (19, 20). As an important part of the central nervous system, the spinal cord has reflex, regulatory, motor and conduction functions. After spinal cord injury, patients may develop motor dysfunction in upper and lower limbs, making them unable to carry out daily activities, which seriously affects the patient's quality of life (21). This study retrospectively analyzed the data of GQOL-74 scores including psychological, social, physical and material functions before and after nursing in two groups. Results indicated superior scores across psychological, social, physical, and material functions in the structured nursing group, suggesting that structured nursing can improve the quality of life of patients. Nir et al. (22) evaluated the effectiveness of structured nursing intervention in elderly patients with a first stroke at 6 months of rehabilitation, demonstrating that structured nursing intervention was superior to conventional nursing in terms of patients’ functional status, depression, self-perceived health, self-esteem, and dietary compliance. This study is consistent with its conclusions, both prove the superiority of structured nursing. This is mainly attributed to the continuous and comprehensive measures of structured nursing, which enhance communication between medical institutions and family members. It can provide long-term nursing assistance to patients after surgery, benefiting their physical and mental health, boosting their confidence in prognosis and rehabilitation, and enabling them to integrate into social life earlier.

This study also demonstrated that structured nursing intervention contributes to a reduction in postoperative complication rates. Patients with cervical spine fracture and spinal cord injury have a long postoperative rehabilitation period. After spinal cord injury, most patients have varying degrees of motor dysfunction, which further increases the difficulty of rehabilitation treatment. During the rehabilitation period, complications such as deep vein thrombosis of the lower extremities and incision infection are prone to occur. In addition, pressure sores are prone to occur due to patients’ inability to move voluntarily after surgery and long-term skin compression; urinary retention or urinary incontinence often occurs, and urinary system infection can easily occur if the nursing is improper (23, 24). The reason why structured nursing is superior to routine care in reducing postoperative complications in this study may be that structured nursing proposed targeted nursing measures from multiple aspects such as medication guidance, dietary guidance, pain care, safety care, psychological care, cognitive intervention, rehabilitation training, and complication prevention, thereby effectively reducing postoperative complications and neurological function damage in patients and improving the prognosis quality. In addition, the clinical efficacy and nursing satisfaction of patients in the structured nursing group were higher than those in the normal group. The reason for this phenomenon is not difficult to understand. For the patients in the structured nursing group, their negative emotions were well relieved during the rehabilitation process, and they received high-quality and comprehensive care in both physiological and psychological aspects, thus achieving higher clinical efficacy and greater satisfaction with nursing.

## 5 Conclusion

This study has demonstrated that structured nursing is more valuable than routine care in the postoperative rehabilitation process of patients with cervical spine fracture and spinal cord injury. This nursing model can intervene from both physiological and psychological aspects of patients, effectively improving their psychological state, enhancing their physiological functions, reducing the incidence of postoperative complications, and promoting their postoperative outcome. It has certain promotion and application value in clinical practice.

### 5.1 Limitation

However, there are also some limitations: 1. The included studies were all single-center studies, which may have selection bias; 2. The included studies were all retrospective studies, which may have confounding bias; 3. The number of cases included in the study was limited and the observation period was short, so the above deficiencies should be further improved in future studies. 4. As nursing staff, we do not have a dedicated outpatient department to follow up on patients, so this study without follow-up data to demonstrate sustained benefit. 5. We will conduct a prospective study to analyze more follow-up data.

## Data Availability

The original contributions presented in the study are included in the article/Supplementary Material, further inquiries can be directed to the corresponding author.
